# Characterization of patient-derived intestinal organoids for modelling fibrosis in Inflammatory Bowel Disease

**DOI:** 10.1007/s00011-024-01901-9

**Published:** 2024-06-06

**Authors:** Ilaria Laudadio, Claudia Carissimi, Noemi Scafa, Alex Bastianelli, Valerio Fulci, Alessandra Renzini, Giusy Russo, Salvatore Oliva, Roberta Vitali, Francesca Palone, Salvatore Cucchiara, Laura Stronati

**Affiliations:** 1 Department of Molecular Medicine, Sapienza University of Rome, Viale Regina Elena 324, 00161 Rome, Italy; 2 Department of Maternal Infantile and Urological Sciences, Sapienza University of Rome, Viale Regina Elena 324, 00161 Rome, Italy; 3 DAHFMO-Unit of Histology and Medical Embryology, Sapienza University of Rome, Via. A. Scarpa, 16, 00161 Rome, Italy; 4 Laboratory of Biomedical Technologies, Italian National Agency for New Technologies, Energy and Sustainable Economic Development (ENEA), Via Anguillarese 301, 00123 Rome, Italy

**Keywords:** Organoids, Fibrosis, Inflammatory Bowel Disease

## Abstract

**Background and aims:**

Intestinal fibrosis is a common complication of Inflammatory Bowel Disease (IBD), namely Crohn's disease (CD) and ulcerative colitis (UC), but the precise mechanism by which it occurs is incompletely understood hampering the development of effective therapeutic strategies. Here, we aimed at inducing and characterizing an inflammation-mediated fibrosis in patient-derived organoids (PDOs) issued from crypts isolated from colonic mucosal biopsies of IBD pediatric patients and age matched-control subjects (CTRLs).

**Methods:**

Inflammatory-driven fibrosis was induced by exposing CTRL-, CD- and UC-PDOs to the pro-inflammatory cytokine TNF-α for one day, followed by a co-treatment with TNF-α and TGF-β1 for three days. Fibrotic response was proven by analyzing inflammatory and fibrotic markers by RT-qPCR and immunofluorescence. Transcriptomic changes were assessed by RNA-sequencing.

**Results:**

Co-treatment with TNF-α and TGF-β1 caused in CTRL- and IBD-PDOs morphological changes towards a mesenchymal-like phenotype and up-regulation of inflammatory, mesenchymal, and fibrotic markers. Transcriptomic profiling highlighted that in all intestinal PDOs, regardless of the disease, the co-exposure to TNF-α and TGF-β1 regulated EMT genes and specifically increased genes involved in positive regulation of cell migration. Finally, we demonstrated that CD-PDOs display a specific response to fibrosis compared to both CTRL- and UC-PDOs, mainly characterized by upregulation of nuclear factors controlling transcription.

**Conclusions:**

This study demonstrates that intestinal PDOs may develop an inflammatory-derived fibrosis thus representing a promising tool to study fibrogenesis in IBD. Fibrotic PDOs show increased expression of EMT genes. In particular, fibrotic CD-PDOs display a specific gene expression signature compared to UC and CTRL-PDOs.

**Supplementary Information:**

The online version contains supplementary material available at 10.1007/s00011-024-01901-9.

## Introduction

Inflammatory bowel disease (IBD), including the two main forms, Crohn disease (CD) and ulcerative colitis (UC), is a chronic multifactorial disorder typically characterized by relapsing and remitting inflammation of the gastrointestinal tract. The incidence and prevalence of IBD are increasing globally, including childhood, and 25% of IBD cases are diagnosed in pediatric age [1, 2].

Intestinal fibrosis, resulting in luminal narrowing and stricture, is a severe complication of IBD that affects the organ function and the life quality of patients [3–5]. It is estimated that around 50% of CD patients will develop fibrotic strictures or penetrating lesions, and up to 75% will eventually need surgery [6–9]. In addition to the fibrosis in strictures, it has been proposed that a certain degree of fibrosis exists in nearly all CD phenotypes, including the early onset CD [10]. Strictures are infrequent in UC, however, a progressive fibrosis is still reported in strong relation with the severity and chronicity of inflammation [11–14].

The mechanisms underlying fibrogenesis are still unclear but chronic intestinal inflammation is thought to be the principal driver [15, 16]. Persistent inflammation causes the activation of myofibroblasts, the main effector cells that produce excessive extracellular matrix (ECM) enabling the establishment of a fibrogenic environment [17–19]. Activation of myofibroblasts is mediated by various factors such as growth factors and cytokines, principally the fibrotic master regulator Transforming Growth Factor (TGF-β), but also IL-1β, IL-33, IL-13 and IL-17 [3, 15, 17, 18, 20].

Furthermore, the process of epithelial-to-mesenchymal transition (EMT), a cellular trans-differentiation program by which epithelial cells acquire mesenchymal characteristics, has been shown to generate epithelial-derived fibroblasts that are responsible for ECM deposition contributing to fibrogenesis [21]. Increasing evidence has showed a role for EMT in the pathogenesis of IBD-derived intestinal fibrosis [22, 23].

Therapeutic options for intestinal fibrosis remain limited and rely exclusively on anti-inflammatory agents, primarily anti-tumor necrosis factor (TNF) agents, that usually improve inflammation but fail to resolve established fibrosis, indeed, fibrotic changes may persist once inflammation is reduced or eliminated [24, 25]. On the other hand, intestinal fibrosis has also been associated to nonresponse to anti-TNF treatment in CD patients [26]. Specifically, the presence of specific inflammatory fibroblasts in CD lesions correlates with failure of anti-TNF therapy [27, 28].

The lack of licensed antifibrotic agents as well as of specific predictors able of identifying IBD patients at a high risk of developing fibrosis toughly highlights the need to improve the understanding of the cellular mechanisms underlying intestinal fibrosis. Much of the experimental evidence related to fibrogenesis is derived from animal models and cell lines. However, both models have limitations. Briefly, in vitro models lack the vast repertoire of cell types and complexity of living organisms [9, 29], and are often focused on myofibroblasts [30]. Instead, although animal models possess traits that are helpful to investigate specific mechanisms involved in gut fibrosis, they are expensive and do not physiologically represent the human disease [31, 32].

The advent of intestinal organoids, 3-D structures originated from either pluripotent (embryonic or induced pluripotent) or adult tissue resident stem cells, has revolutionized IBD research [33–35].

Intestinal organoids may differentiate from human induced pluripotent stem cells (iPSC) [36] or from human embryonic stem cells [37]. More recently, patient-derived intestinal organoids (PDOs), resulting from stem cells of endoscopically taken mucosal biopsies, retain many functions of the intestinal crypts/epithelium, such as supporting self-renewal, self-organization, barrier function, and differentiation into epithelial cell subtypes, thus, representing a very promising in vitro model to deeply investigate the pathophysiology of IBD, including the onset and progression of fibrosis [38–42].

Here, we explore the potential of PDOs derived from pediatric with CD and UC patients and from age-matched controls (CTRLs) to be used as a reliable model of intestinal fibrosis.

## Materials and methods

### Patients

Pediatric patients with CD (n = 3) and UC (n = 3) were recruited for this study from November 2020 through May 2021 during routine colonoscopy at the Pediatric Gastroenterology and Liver Unit of the Department of Maternal Infantile and Urological Sciences, Sapienza University of Rome-University Hospital Umberto I. All patients had an established diagnosis of IBD in accordance with the ESPGHAN Porto Criteria [43]. In addition, pediatric patients undergoing endoscopy for functional gastrointestinal complaints but with grossly normal endoscopies were included as controls (CTRLs; n = 3). Table 1 shows patient cohort characteristics.

**Table 1:** Patient cohort characteristics

Subjects	Sex	Age	Age at diagnosis	Therapy (duration)
CTRL1	F	11		–
CTRL2	M	10		–
CTRL3	M	13		–
CD1	F	17	8	Corticosteroids, biologics (anti-TNF) (8 years)
CD2	M	13	12	Mesalazine, corticosteroids, biologics (anti-TNF), antibiotics (1 year)
CD3	M	14	9	Biologics (anti-TNF) (6 years)
UC1	F	14	12	Corticosteroids, mesalazine, immunosuppressive, antibiotics, Proton Pump Inhibitor (1 month)
UC2	F	15	14	Mesalazine, corticosteroids (2 months)
UC3	F	13	10	Mesalazine (1 year)

All patients or caregivers gave written informed consent before sample collection (approved by the Ethics Committee of the Policlinico Umberto I Hospital, EC No. 4771/2018).

### PDO isolation and culture

PDOs were generated from isolated crypts of mucosal biopsies of macroscopically non-inflamed transverse colon. Biopsies were collected in Advanced DMEM/F12 (GIBCO) supplemented with 10 mM HEPES (Sigma-Aldrich), 2 mM L-Glutamine (CORNING), 100 units/ml and 100 μg/ml Penicillin/Streptomycin (CORNING), and then washed with cold PBS for five times. Next, the biopsies were incubated in 12.5 mM EDTA/PBS at + 4 °C for 1.5 h with shaking. Biopsies were vigorously resuspended in 10 ml of cold PBS to isolate intestinal crypts, and the supernatant fraction was collected. This process was repeated four times to attain 40 ml of PBS containing crypts to which 10 ml of FBS were added. Isolated crypts were centrifuged at 1300 rpm for 5 min and resuspended in 5 ml of Advanced DMEM/F12 supplemented with 100 units/ml and 100 μg/ml Penicillin/Streptomycin, 10 mM HEPES, and 2 mM L-Glutamine. Crypts were then centrifuged at 1000 rpm for 5 min. Supernatant was removed and the pellet was re-suspended into Matrigel™ (CORNING) with Advanced DMEM/F12 medium (1:1 ratio) and plated into 24 well plates. PDOs were cultured in 50% Advanced DMEM/F12, supplemented with 20% FBS, 500 nM A-8301 (Sigma-Aldrich), 10 μM SB-202190 (Sigma-Aldrich), 10 mM Nicotinamide (Sigma-Aldrich), 1.25 mM N-Acetylcysteine (Sigma-Aldrich), 50 ng/ml EGF (Life Technologies), B-27 supplement (Life Technologies), and 50% of conditional medium produced from L-WRN cell line (ATCC, #CRL-3276) engineered to secrete Wnt3a, R-spondin 3, and Noggin in the medium [44]. Medium was replaced every 2–3 days and passaging of PDOs was performed by mechanically dissociation every 4 days with a 1:2 split ratio.

### PDO treatments

Organoids were seeded with a 1:2 split ratio in a 24-well plate. After plating, PDOs were cultured for 24 h in IntestiCult™ Organoid Growth Medium (Human) (STEMCell Technologies). The day after, PDOs were exposed to 100 ng/ml TNF-α (PeproTech) in IntestiCult™ Organoid Growth Medium for 24 h and subsequently treated with a combination of 100 ng/ml TNF-α and 50 ng/ml TGF-β1 (Abcam) in IntestiCult™ Organoid Growth Medium for further 72 h. Cytokine-containing medium was replaced every 2 days.

### PDO immunofluorescence

PDOs (CD n = 3; UC n = 3 and CTRLs n = 3) were embedded in OCT according to Zhang protocol [45]. Then, 20 μm thick organoids sections were made using a cryostat (Leica Microsystems) and subsequently mounted on positively charged adhesive slides (Epredia).

For immunofluorescence staining, the frozen section slides were fixed for 5 min in 4% Paraformaldehyde and then permeabilized for 30 min with 0.2% TritonX-100 in PBS. The slides were then incubated at room temperature for 20 min in 3% BSA in PBS. Primary antibodies were as follows: anti-Fibronectin (Abcam), anti-Vimentin (BD Biosciences) and anti-E-Cadherin (Thermo Fischer Scientific). Primary antibodies were diluted in 0.5% BSA in PBS, applied to organoids section and incubated at 4 °C, overnight. The day after, the slides were incubated for 1 h at room temperature with the secondary antibodies (anti-rabbit Alexa Fluor 488; anti-mouse Alexa Fluor 488; anti-rabbit Alexa Fluor 633, Invitrogen) diluted in a 0.5% BSA in PBS. The slides were then incubated for 5 min with DAPI (Thermo Fischer Scientific) and finally covered with a drop of mounting medium.

Images were acquired using the Zeiss LSM900 Airyscan 2 confocal microscope. Optical spatial series with a step size of 1 μm were recovered.

### RNA isolation and sequencing

Matrigel-embeded PDOs were lysed in 500 μl Trizol (LifeTechnologies) and total RNA was isolated with Direct-zol RNA MiniPrep kit (ZymoResearch) following manufacturer’s instructions. For Total RNA-Seq, library preparation and sequencing were performed by Procomcure Biotech GmbH (Austria). The library preparation was performed using Nextflex Rapid Directional RNA-Seq kit 2.0 Kit (PerkinElmer) with Nextflex Ribonaut rRNA depletion kit (PerkinElmer). Libraries were sequenced using Illumina NovaSeq 6000 with 2 × 150 bp paired-end run; Seq throughput: 50 M PE reads.

### RT-qPCR

One hundred ng of RNA were reverse transcribed by ProtoScript II Reverse Transcriptase (New England BioLabs) using random primers. RT-qPCR was performed using GoTaq® qPCR Master Mix. Primer sequences are as follow: *COL4A1* Fw 5′-GGTGTTGCAGGAGTGCCAG-3′ and Rev 5′-GCAAGTCGAAATAAAACTCACCAG-3′; *FN1* Fw 5′-AGACCATACCTGCCGAATGTAG-3′ and Rev 5′-GAGAGCTTCCTGTCCTGTAGAG-3′; *GAPDH* Fw 5′-GAAATCCCATCACCATCTTCCAGG-3′ and Rev 5′-GAGCCCCAGCCTTCTCCATG-3′; *SERPINE1* Fw 5′-CACAAATCAGACGGCAGCAC-3′ and Rev 5′-GGGCGTGGTGAACTCAGTATAG-3′; *ACTA2* Fw 5′CCGACCGAATGCAGAAGGA-3′ and Rev 5′-ACAGAGTATTTGCGCTCCGAA-3′; *IL1B* Fw 5′-AGCTCGCCAGTGAAATGATGG-3′ and Rev 5′-GTCCTGGAAGGAGCACTTCAT-3′, *SNAI2* Fw 5′-GCCAAACTACAGCGAACTGGA-3′ and Rev 5′-ACAGAGTATTTGCGCTCCGAA-3′. The expression level of each gene was assessed using the 2^−(∆∆Ct)^ method, and GAPDH was used for the normalization.

### Bioinformatic analysis

Fastq data were trimmed (cutadapt v 4 [46]). Trimmed reads were aligned to the hg38 version of the human genome with hisat v2.2.1 [47] and Duplicate reads were filtered using samtools [48]. Gene level read counts were computed using htseq-count (v0.12.4) [49] with gencode v42 annotation [50]. Genes labeled as "pseudogene" were removed from the gtf file before running htseq-count.

Raw counts have been analyzed using DESeq2 [51]. Adjusted P-values were computed using the results function of the DESeq2 package, choosing the "BH" algorithm [52].

Functional annotation (GO-term) and enrichment analyses were performed using DAVID (2021 update) [53].

### Data availability

Fastq data cannot be shared publicly for the privacy of individuals that participated in the study. The data will be shared on reasonable request to the corresponding author. Gene counts are available in the article and in its online supplementary material (Supplementary Table 1).

### Statistics

The results of RT-qPCR are expressed as the mean ± SEM. Comparison between treated and untreated PDOs were performed by paired *t*-test. A *p* value ≤ 0.05 was considered statistically significant.

## Results

### Co-treatment with TNF-α and TGF-β1 induces a fibrotic phenotype in PDOs

**Fig. 1:**
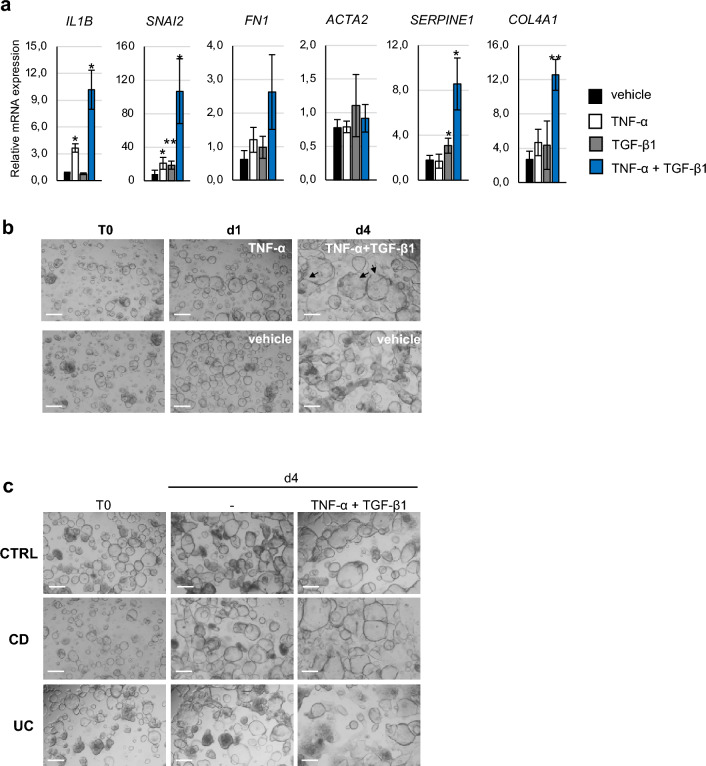
Co-treatment with TNF-α and TGF-β1 induces an EMT-like molecular and morphological phenotype in intestinal PDOs. PDO cultures were established from intestinal crypts isolated from colon biopsies of healthy pediatric subjects. PDOs were cultured for 4 days with TNF-α (100 ng/ml), or for 3 days with TGF-β1 (50 ng/ml), or for 24 h with TNF-α (100 ng/ml) followed by a cocktail of TNF-α (100 ng/ml) and TGF-β1 (50 ng/ml) for subsequent 3 days (TNF-α + TGF-β1), or with vehicle. **a** The expression of inflammatory (*IL1B*), mesenchymal (*SNAI2*), and fibrotic (*FN1, ACTA2, SERPINE1* and *COL4A1*) genes were assessed by RT-qPCR. Data are expressed as mean ± SEM. * = p-value ≤ 0.05; ** = p-value ≤ 0.01 n = 3. **b** Morphological changes were documented by light microscopy at day 1 (d1) upon 24 h with TNF-α (100 ng/ml) and following 3 days with TNF-α + TGF-β1 (day 4; d4). **c** PDO from CTRLs, CD and UC pediatric patients were cultured for 24 h with TNF-α (100 ng/ml) followed by a cocktail TNF-α (100 ng/ml) and TGF-β1 (50 ng/ml) for subsequent 3 days (TNF-α + TGF-β1) or with vehicle (-). Morphological changes were documented by light microscopy. Arrows indicate organoids that lose their integrity with cells migrating out. Scale bars correspond to 200 µm

In IBD, chronic inflammation triggers fibrosis [15, 16]. We initially set-up a protocol for mimicking an inflammatory-driven fibrosis in PDOs derived from intestinal crypts of control subjects. PDOs were treated with TNF-α (4 days) alone or with TGF-β1 (3 days) alone or with TNF-α (24 h) followed by a co-treatment with TNF-α and TGF-β1 (3 days). The expression levels of inflammatory (*IL1B*, namely IL-1β), mesenchymal (*SNAI2*, also known as SLUG), and fibrotic (*FN1*, *ACTA2*, also known as αSMA, *SERPINE1* and *COL4A1*) genes were assessed by RT-qPCR (Fig. 1a). Our data highlight that TNF-α or TGF-β1 alone slightly stimulated *IL1B* and *SNAI2* or *SNAI2* and *SERPINE1*, respectively. Interestingly, treatment with the TNF-α and TGF-β1 cocktail induced a more evident response characterized by a significant upregulation of *IL1B*, *SNAI2*, *SERPINE1* and *COL4A1* in CTRL-PDOs. Coherently, the morphological analysis revealed that the co-treatment with TNF-α and TGF-β1 caused phenotypical changes in organoid structure typical of EMT, with spheroid structures that lose their integrity because of cells that migrate out, suggesting the acquisition of a mesenchymal phenotype (Fig. 1b). We concluded that co-treatment with TNF-α and TGF-β1 triggers a fibrotic response in intestinal CTRL-PDOs.

Based on previous results, we next exposed PDOs of CD and UC patients only to TNF-α and TGF-β1 co-treatment. Accordingly, morphological changes were comparable to those observed in CTRL-PDOs, showing a loss of structural integrity, above all in UC-PDOs, and the acquisition of mesenchymal phenotype (Fig. 1c).

To further stress the potency of the cytokine co-treatment in inducing overt fibrosis, as compared to single treatment, IBD PDOs were exposed to TNF-α or TGF-β1 alone as well as to co-treatment with cytokines and *IL1B*, *SNAI2*, *ACTA2*, *COL4A1*, *SERPINE1* and *FN1* were analyzed by RT-qPCR. Results showed that the cytokine co-exposure induced the highest levels of *IL1B*, *SNAI2*, *SERPINE1* and *COL4A1* (Supplementary Figure S1).

**Fig. 2:**
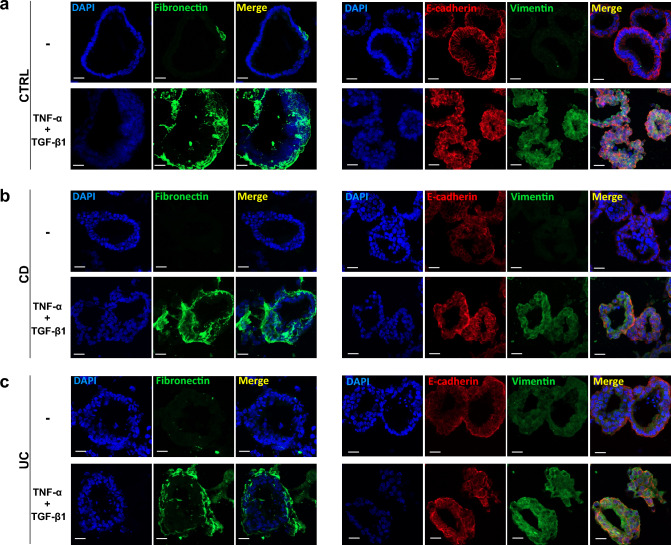
Treatment with TNF-α and TGF-β1 stimulates the expression of FN1 and Vimentin in CTRL- and IBD-PDOs. Expression of FN1 (green, left panels), Vimentin (green, right panels) and E-cadherin (red, right panels) was visualized by immunofluorescence in CTRL-, CD- and UC-PDOs treated or not with TNF-α and TGF-β1. Scale bars correspond to 20 µm

Moreover, the levels of fibrotic markers, Fibronectin (FN1) and vimentin, and of the epithelial marker E-cadherin were also analyzed by immunofluorescence and examined by confocal microscopy. Images showed that FN1 and vimentin, weakly expressed in untreated CTRL and IBD PDOs, are toughly increased after treatment with TNF-α and TGF-β1. Differently, we didn’t observe differences in E-Cadherin expression (Fig. 2).

We concluded that the co-exposure to the inflammatory cytokine TNF-α and the pro-fibrotic cytokine TGF-β1 is the most efficient treatment to induce active fibrosis in PDOs. Therefore, from this moment on, PDOs were always treated with double exposure.

### Transcriptomic analysis in PDOs derived from CTRLs, CD and UC patients reveals that TNF-α and TGF-β1 prompt fibrosis by upregulating EMT genes

To analyze and compare the transcriptional changes in PDOs from CTRLs, CD and UC patients upon co-treatment with TNF-α and TGF-β1, we performed a total RNA Sequencing.

**Fig. 3:**
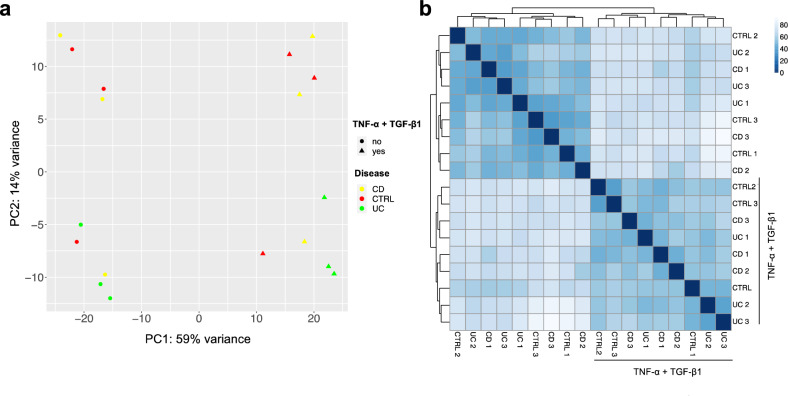
Transcriptomic profile after exposure to TNF-α and TGF-β1 is shared by CTRL-, CD- and UC-PDOs. CTRL-, CD- and UC-PDOs treated for 24 h with TNF-α (100 ng/ml) followed by a cocktail of TNF-α (100 ng/ml) and TGF-β1 (50 ng/ml) for subsequent 3 days (TNF-α + TGF-β1) or with vehicle were analyzed by total RNA-seq. PCA **a** and Euclidean distance matrix of the samples **b** revealed that major transcriptional variations are driven by TNF-α and TGF-β1 treatment, regardless to the disease

Principal component analysis (PCA) revealed that major transcriptional variations among samples (PC1, 59% explained variance) are driven by treatment with segregation between treated and untreated PDOs (Fig. 3a). Then, we computed a Euclidean distance matrix of the samples and found that, coherently with PCA results, the groups segregated by treatment and not by disease (Fig. 3b). These data evidenced robust and reproducible effects of cytokine treatment in modulating gene expression in all intestinal PDOs, regardless of the disease.

**Fig. 4:**
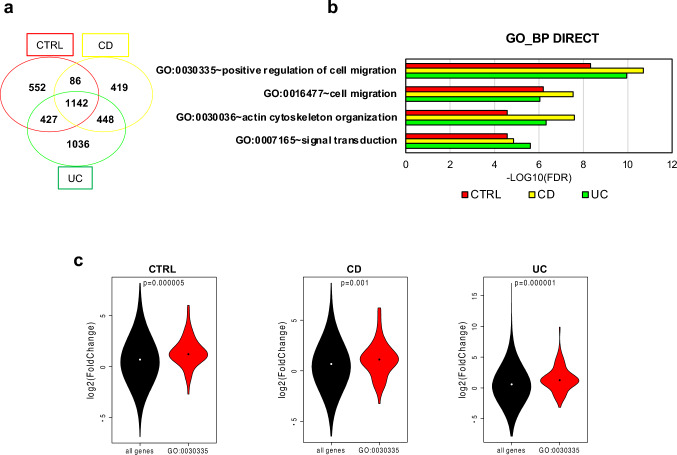
TNF-α and TGF-β1 modulate the expression of genes involved in EMT and specifically up-regulate genes that stimulate cell migration. DEGs (adj P-value < 0.05) between TNF-α and TGF-β1 treated PDOs and their controls were identified in CTRL- (in red), CD- (in yellow) and UC-PDOs (in green) and the overlap was depicted as a Venn diagram (**a**). Four out of 5 top-scored GO terms in the Biological Process (BP) Direct category were shared in the three lists of DEGs (b) and pinpointed to EMT-related pathways. Violin plots displayed the distribution of the log2 Fold change of DEGs belonging to the GO:0030335 ~ positive regulation of cell migration as compared to all the other genes (**c**)

Therefore, we compared the differentially expressed genes (DEGs, adj P-value < 0.05) between CTRLs and CTRLs TNFα + TGFβ1 (2207 DEGs), or between CD and CD TNFα + TGFβ1 (2095 DEGs), or between UC and UC TNFα + TGFβ1 (3053 DEGs) (Supplementary tables S2–4). A Venn diagram showed that the highest number of DEGs upon treatment were common to all PDOs, suggesting that they were altered by the occurrence of inflammatory-driven fibrosis nevertheless the presence of the disease (Fig. 4a).

To get insights in the biological function of DEGs, we performed GO term enrichment analysis using DAVID[53]. We found that the 5 most enriched GO terms in the Biological Process (BP) category were highly overlapping in the three pairwise comparisons (CTRLs vs CTRLs TNF-α + TGF-β1; CD vs CD TNF-α + TGF-β1; and UC vs UC TNF-α + TGF-β1; Supplementary tables S5–7) and pinpointed to EMT-related processes, *i.e*. GO:0030335 ~ positive regulation of cell migration, GO:0016477 ~ cell migration, GO:0030036 ~ actin cytoskeleton organization and GO:0007165 ~ signal transduction (Fig. 4b).

We focused on genes belonging to the top-scored shared GO term, namely GO:0030335 ~ positive regulation of cell migration, wondering if they were upregulated by TNF-α and TGF-β1 co-treatment. The volcano plots of DEGs in the three pairwise comparisons as above (Supplementary Figure S2) showed that in each comparison most DEGs belonging to GO:0030335 were upregulated. Coherently, we found that the distribution of the log2 Fold Change of DEGs belonging to GO:0030335 was significantly shifted towards positive values compared to all other genes (Fig. 4c), confirming that TNF-α and TGF-β1 treatment increased the expression of genes involved in positive regulation of cell migration.

In conclusion, the transcriptomic profile of CTRL- and IBD-PDOs demonstrated that fibrotic stimuli modulate the expression of genes involved in EMT and specifically increased genes involved in the positive regulation of cell migration.

### Intestinal PDOs derived from CD patients display a specific signature in response to fibrotic stimuli

**Fig. 5:**
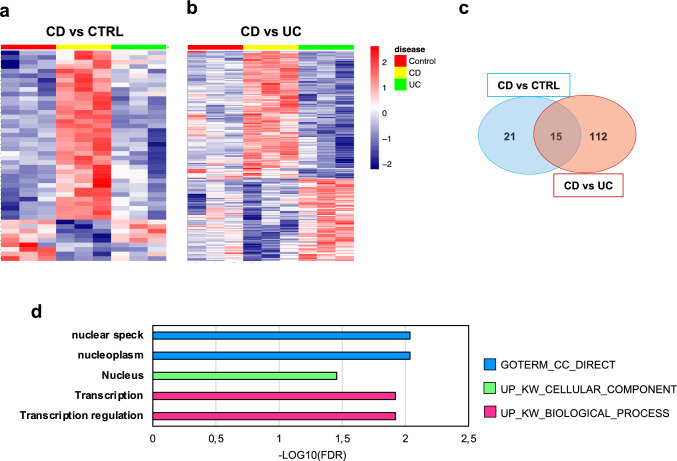
PDOs derived from CD patients display a specific response to fibrotic stimuli. Heatmaps depicting: **a** the 46 genes that are differentially expressed between CD-PDOs treated with TNF-α and TGF-β1 and CTRL-PDOs treated with TNF-α and TGF-β1 (CD vs CTRL); **b** the 210 genes that are differentially expressed between CD-PDOs treated with TNF-α and TGF-β1 and CTRL-PDOs treated with TNF-α and TGF-β1 (CD vs UC). Genes specifically upregulated in fibrotic CD-PDOs as compared to CTRL-, and UC-PDOs or both are displayed as a Venn diagram (**c**). Functional enrichment analysis of these genes highlighted annotations pinpointing to nuclear localization and transcription regulation (**d**)

As mentioned above, a significant percentage of CD patients (up to 50%) ultimately progress to fibrostenosis [54], while the rate of stenosis is much lower (range: 1%–11%) in UC patients [55]. Hence, we investigated the possibility that PDOs could be helpful in understanding the higher prevalence of fibrosis in CD. Thus, the whole transcriptome of CD- and CTRL- or UC-PDOs after treatment with TNF-α and TGF-β1 were compared (adj P-value < 0.05). Forty-six DEGs between treated CD- and CTRL- PDOs (Supplementary table S8), and 210 DEGs between treated CD- and UC- PDOs were identified (Supplementary table S9). Relative expression of DEGs in CD vs CTRLs (Fig. 5a, gene expression in UC is also shown) and in CD vs UC (Fig. 5b, gene expression in CTRLs is also shown) is reported as heatmaps showing that most of these genes are specifically overexpressed in fibrotic CD- as compared to both CTRL- and UC-PDOs. Remarkably, 36 and 127 genes were up-regulated in CD vs CTRLs and in CD vs UC, respectively, with an overlap of 15 genes (Fig. 5c). Overexpressed genes were pooled and a functional annotation and enrichment analyses taking advantage of DAVID was performed.

Results indicated a statistically significant enrichment only in the GO term category Cell Component (CC_DIRECT) and in the UniprotKB Keywords (UP_KW) types Cellular Component and Biological Process, pinpointing to nuclear localization and involvement in transcription regulation of genes upregulated in fibrotic CD (Fig. 5d, Supplementary Table S10).

Overall, these findings suggest that CD-PDOs exhibit a specific gene expression signature when exposed to fibrotic stimuli, mainly characterized by the upregulation of nuclear factors controlling transcription.

## Discussion

Fibrosis is a condition common to many clinical disorders that contributes to over 35% of all deaths worldwide [56]. Fibrosis is characterized by excessive ECM accumulation in damaged tissues that can ultimately cause failure in many organs.

Specifically, intestinal fibrosis is a usual complication in IBD, especially in CD patients. Although the consequences of fibrosis in other organs are well documented, this phenomenon has not yet been thoroughly explored in the gut where fibrosis is only just beginning to attract interest.

Strong evidence indicates that chronic inflammation triggers fibrosis that, once established, may progress independently. However, the comprehension of pathogenetic mechanisms leading to gut fibrosis is still in its infancy, thus, it is mandatory to expand the knowledge on this issue as well as on the associations between organ fibrosis and the underlying molecular pathways or functions.

The study of intestinal fibrogenesis requires valuable experimental models capable of recapitulating the steps of its development and progression. PDOs derived from intestinal crypts isolated from endoscopy biopsies retain the genetic and transcriptomic profile of the tissue of origin over time [35, 39], thus offering a unique and direct link to the disease.

Here, we use for the first time PDOs of CD, UC and age-matched control subjects to set up a protocol to induce an overt fibrosis. Since it is well known that in IBD TNF-α and TGF-β1 are the master regulators of inflammation [57] and fibrosis [58], respectively, we exposed PDOs to treatments with each cytokine alone or both of them in order to reproduce at its best an inflammatory-driven fibrosis.

Results clearly showed that the co-treatment with TNF-α and TGF-β1 induced a reproducible fibrotic response in all patient and control PDOs, regardless of the occurrence of the disease, that was more robust than the single treatment. Fibrosis was proven both phenotypically and molecularly. Indeed, the treatment caused evident phenotypical changes in organoid structure that lose their integrity due to the migration of epithelial cells that acquired a mesenchymal phenotype as confirmed by the immunofluorescence staining showing the expression of the mesenchymal markers FN1 and Vimentin. However, cells did not decrease the level of the epithelial marker E-cadherin, but this was not surprising as previous data demonstrated in different experimental models that intestinal fibrosis is characterized by the presence of epithelial cells undergoing to EMT that co-express epithelial (E-cadherin and cytokeratin) and mesenchymal markers (α-SMA and Vimentin) [23, 59, 60].

Further, treated PDOs highly expressed mRNA levels of mesenchymal and fibrotic markers such as SNAI2, ACTA2, COL4A1, SERPINE1 and FN1.

All these evidences allowed to conclude that the exposure to the mix TNF-α/TGF-β1 was the most efficient treatment to induce active fibrosis in PDOs, thus it was adopted for all subsequent experiments.

Then, a total RNA sequencing was performed in order to compare the transcriptomic profile in fibrotic and non-fibrotic CD, UC and control PDOs. Data analysis indicated that experimental groups segregated by treatment and not by disease, confirming previous evidence that all PDOs were altered by the occurrence of inflammatory-driven fibrosis undeterred by the presence of the disease. Interestingly, the analysis of the biological function of differential expressed genes between treated and untreated PDOs highlighted that genes were mainly involved in cell migration. This evidence was in agreement with the phenotypical changes previously observed in fibrotic organoids indicating a loss of cell adhesion between cells and an increased migratory activity compatible with the acquisition of a mesenchymal fibroblast-like phenotype.

It is known that type-2 EMT is associated with wound healing, tissue regeneration and organ fibrosis. During organ fibrosis, type-2 EMT occurs as a reparative-associated process in response to ongoing inflammation and eventually leads to organ destruction [61]. In the context of type-2 EMT, epithelial cells gain motility and migrate to the site of injury to participate to tissue integrity restoration [62]. Accordingly, in this study, the transcriptomic profile of CTRL and IBD PDOs demonstrated that fibrotic stimuli modulate the expression of genes involved in EMT and specifically increase genes involved in the positive control of cell migration.

Gut fibrosis is differently expressed in CD and UC. Indeed, it results in stricture formation and obstruction in CD and increased wall stiffness leading to symptoms in UC [63]. Besides, up to 21% of patients with CD present with strictures at diagnosis, while the rate of stenosis varies from 1 to 11% in UC [54]. Given these differences, the molecular mechanisms that regulate the onset and development of fibrosis in the two disorders are still poor or completely misunderstood. Hence, with the aim of highlighting any specificities, RNA-seq data were further analyzed and 210 differentially expressed genes were identified comparing treated CD- and UC- PDOs. Intriguingly, most of these genes were specifically overexpressed in fibrotic CD PDOs and were mainly involved in transcription regulation. To sum up, this finding revealed that CD-PDOs display a specific gene expression signature when exposed to fibrotic stimuli, mainly characterized by the upregulation of nuclear factors controlling transcription.

Although further experiments are required to uncover the role of these genes in the context of intestinal fibrogenesis, however, we here demonstrate for the first time that epithelial cells arising from the intestinal mucosa of CD patients react differently to fibrotic stimuli as compared to UC.

Even though PDOs do not mimic the complexity of the in vivo situation because of the lack of the immune system compartment and the mesenchymal niche, however, they allow to specifically dissect how the intestinal epithelium participates in the development and progression of fibrosis. This study actually demonstrates that intestinal PDOs represent a promising novel platform for the study of EMT in the onset and progression of fibrosis in IBD patients. Moreover, our data pave the way to the use of IBD-PDOs as an ex vivo model to explore the anti-inflammatory and anti-fibrotic effects of new drugs and to analyze individual patient disease, prompting the design of personalized treatments.

### Supplementary Information

Below is the link to the electronic supplementary material.

Supplementary file1 (PDF 630 KB)

Supplementary file2 (XLSX 4125 KB)

Supplementary file3 (XLSX 228 KB)

Supplementary file4 (XLSX 212 KB)

Supplementary file5 (XLSX 108 KB)

Supplementary file6 (XLSX 64 KB)

Supplementary file7 (XLSX 69 KB)

Supplementary file8 (XLSX 87 KB)

Supplementary file9 (XLSX 14 KB)

Supplementary file10 (XLSX 30 KB)

Supplementary file11 (XLSX 11 KB)

## Data Availability

The data underlying this article cannot be shared publicly for the privacy of individuals that participated in the study. The data will be shared on reasonable request to the corresponding author.
