# Supplementary material for: Characterization of patient-derived intestinal organoids for modelling fibrosis in Inflammatory Bowel Disease
Source: Inflamm Res. 2024 Jun 6;73(8):1359–70. doi: 10.1007/s00011-024-01901-9 (PMC11282153; doi:10.1007/s00011-024-01901-9)
Supplement: Supplementary file 1 — Supplementary file1 (PDF 630 KB) [file 11_2024_1901_MOESM1_ESM.pdf]

Supplementary Figure S1

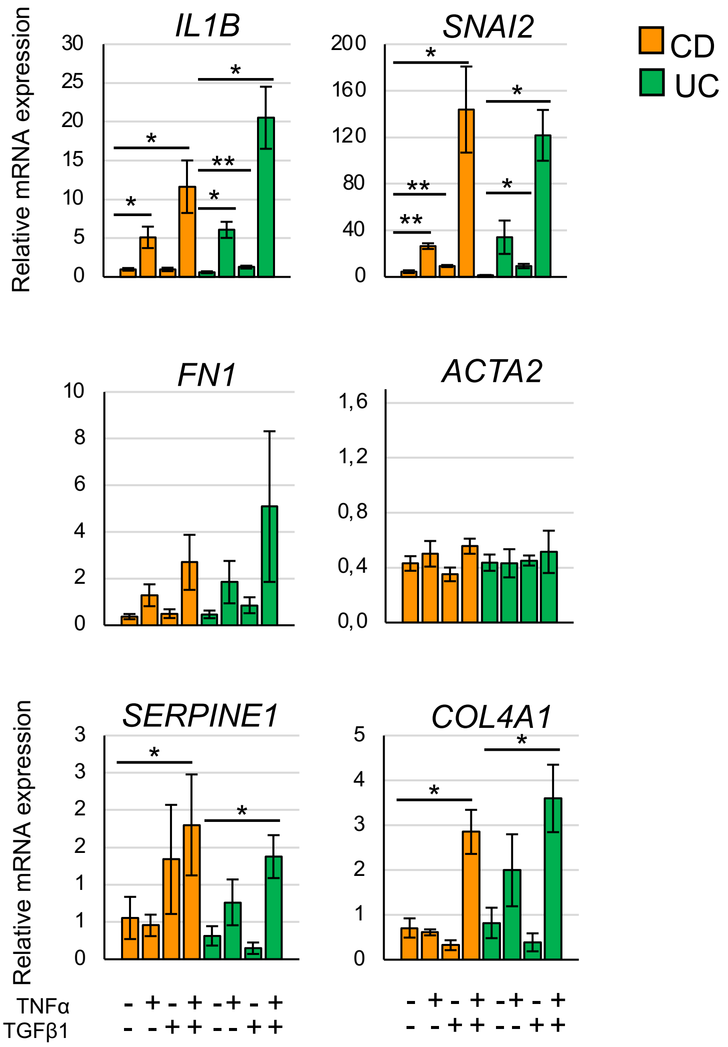

Supplementary Figure S1. The combination of TNF-α and TGF-β1 is more efficient than single cytokines in stimulating inflammatory, mesenchymal and fibrotic markers in IBD-PDOs

CD- and UC-PDOs were cultured for 4 days with TNF-α (100 ng/ml), for 3 days with TGF-β1 (50 ng/ml), for 24 hours with TNF-α (100 ng/ml) followed by a cocktail TNF-α (100 ng/ml) and TGF-β1 (50 ng/ml) for subsequent 3 days (TNF-α +TGF-β1) or with vehicle. The expression of inflammatory (*IL1B*), mesenchymal (*SNAI2*), and fibrotic (*FN1*, *ACTA2*, *SERPINE1* and *COL4A1*) genes were assessed by RT-qPCR. Data are expressed as mean ± SEM. * = p-value ≤ 0.05; ** = p-value ≤ 0.01 n = 3.

Supplementary Figure S2

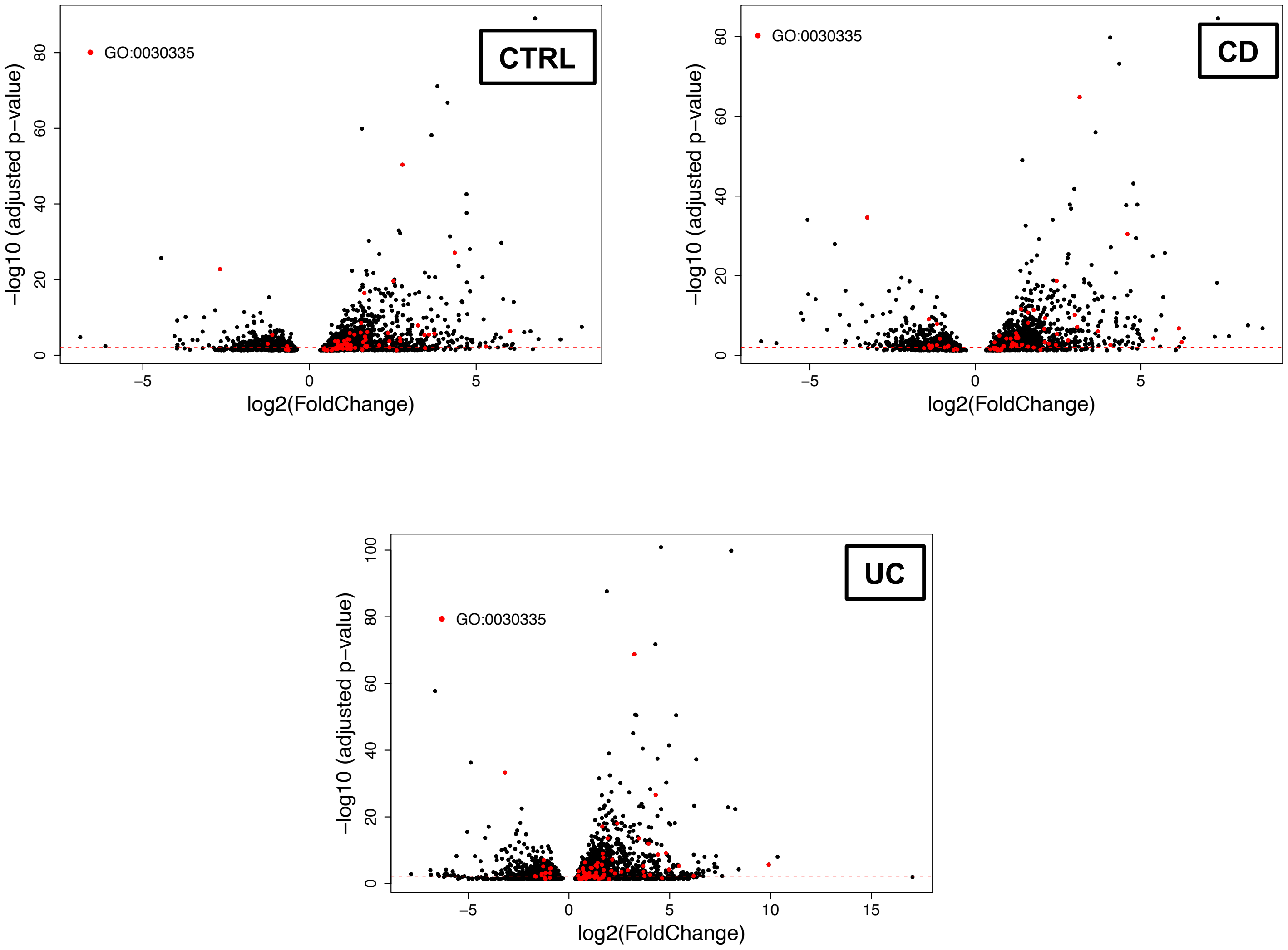

Supplementary Figure S1. TNF-α and TGF-β1 up-regulate genes that positively control cell migration.

Volcano plots display DEGs (adj P-value < 0.05) between TNF-α and TGF-β1 treated PDOs and its controls in CTRL-, CD- and UC-PDOs. DEGs belonging to the *GO:0030335~positive regulation of cell migration* are highlighted in red.

Supplementary table legend.

Supplementary table 1. List of RNA-seq gene counts for each sample.

Supplementary table 2. DEGs (adj P-value < 0.05) between CTRL and CTRL (TNFα + TGFβ1) PDOs

Supplementary table 3. DEGs (adj P-value < 0.05) between CD and CD (TNFα+TGFβ1) PDOs

Supplementary table 4. DEGs (adj P-value < 0.05) between UC and UC (TNFα+TGFβ1) PDOs

Supplementary table 5. Gene Ontology (GO) term enrichment analysis of DEGs between CTRL and CTRL (TNFα + TGFβ1) PDOs (FDR< 0.05).

Supplementary table 6. Gene Ontology (GO) term enrichment analysis of DEGs between CD and CD (TNFα+TGFβ1) PDOs (FDR< 0.05).

Supplementary table 7. Gene Ontology (GO) term enrichment analysis of DEGs between UC and UC (TNFα+TGFβ1) PDOs (FDR< 0.05).

Supplementary table 8. DEGs (adj P-value < 0.05) between CD-PDOs treated with TNFα and TGFβ1 and CTRL-PDOs treated with TNFα and TGFβ1.

Supplementary table 9. DEGs (adj P-value < 0.05) between CD-PDOs treated with TNFα and TGFβ1 and UC-PDOs treated with TNFα and TGFβ1.

Supplementary table 10. Functional annotation and enrichment using DAVID Knowledgebase v2023q3 (FDR< 0.05) of genes upregulated in fibrotic CD-PDOs as compared to fibrotic CTRL-, and UC-PDOs or both.
